# Superior mesenteric artery-related outcomes in fenestrated/branched endografting for complex aortic aneurysms

**DOI:** 10.3389/fcvm.2023.1252533

**Published:** 2023-09-13

**Authors:** E. Gallitto, G. Faggioli, A. Vacirca, M. Lodato, A. Cappiello, A. Logiacco, F. Feroldi, R. Pini, M. Gargiulo

**Affiliations:** ^1^ Vascular Surgery, University of Bologna—DIMEC, Bologna, Italy; ^2^ Vascular Surgery Unit, IRCCS, University Hospital Policlinico S. Orsola, Bologna, Italy

**Keywords:** superior mesenteric artery (SMA), thoracoabdominal aneurysm repair, complex aortic aneurysm, fenestrated endograft, branched endograft

## Abstract

**Aim:**

Early/follow-up durability of superior mesenteric artery (SMA) stent-grafts is crucial after fenestrated/branched endografting (FB-EVAR) in complex abdominal aortic aneurysms (CAAAs) and thoracoabdominal aortic aneurysms (TAAAs). The study aimed to report early/midterm outcomes of SMA incorporated during FB-EVAR procedures.

**Methods:**

FB-EVAR procedures performed between 2016 and 2021 in a single institution were reviewed. Anatomical SMA characteristics were analyzed. The SMA configuration was classified into three types according to the angle between the SMA main trunk and the aorta: (A) perpendicular, (B) downward, and (C) upward. SMA-related technical success (SMA-TS: cannulation and stenting, patency at completion angiography without endoleak, stenosis/kinking, dissection, bleeding, and 24-h mortality) and SMA-adverse events (SMA-AEs: one among bowel ischemia, stenosis, occlusion, endoleak, reinterventions, or SMA-related mortality) were assessed.

**Results:**

Two hundred FB-EVAR procedures with SMA as the target artery were performed. The indication for FB-EVAR was CAAAs and TAAAs in 99 (49%) and 101 (51%) cases, respectively. The SMA configuration was A, B, and C in 132 (66%), 63 (31%), and 5 (3%) cases, respectively. SMA was incorporated with fenestrations and branches in 131 (66%) and 69 (34%) cases, respectively. Directional branch (*P* < .001), aortic diameter ≥35 mm at the SMA level (*P* < .001), and ≥2 SMA bridging stent-grafts (*P* = .001) were more frequent in TAAAs. Relining of the SMA stent-graft with a bare metal stent was necessary in 41 (21%) cases to correct an acute angle between the stent-graft and native artery (39), stent-graft stenosis (1), or SMA dissection (1). Relining was associated with type A or C SMA configuration (OR: 17; 95% CI: 1.8–157.3; *P* = .01). SMA-TS was achieved in all cases. Overall, 15 (7.5%) patients had SMA-AEs [early: 9 (60%), follow-up: 6 (40%)] due to stenosis (2), endoleak (8), and bowel ischemia (5). Aortic diameter ≥35 mm at the SMA level was an independent risk factor for SMA-AEs (OR: 4; 95% CI: 1.4–13.8; *P* = .01). Fourteen (7%) patients died during hospitalization with 10 (5%) events within the 30-postoperative day. Emergency cases (OR: 33; 95% CI: 5.7–191.3; *P* = .001), peripheral arterial occlusive disease (OR: 14; 95% CI: 2.3–88.8; *P* = .004), and bowel ischemia (OR: 41; 95% CI: 1.9–87.9; *P* = .01) were risk factors for 30-day/in-hospital mortality. The mean follow-up was 32 ± 24 months; estimated 3-year survival was 81%, with no case of late SMA-related mortality or occlusion. The estimated 3-year freedom from overall and SMA-related reinterventions was 74% and 95%, respectively.

**Conclusion:**

SMA orientation determines the necessity of stent-graft relining. Aortic diameter ≥35 mm at the SMA level is a predictor of SMA-AEs. Nevertheless, SMA-related outcomes of FB-EVAR are satisfactory, with excellent technical success and promising clinical outcomes during the follow-up.

## Introduction

Fenestrated and branched endografting (FB-EVAR) is an established technique for the endovascular treatment of complex abdominal aortic aneurysms (CAAAs: juxta/pararenal aneurysms) and thoracoabdominal aortic aneurysms (TAAAs) where anatomically feasible, and particularly in patients at high risk for open repair (1). Single- and multicenter experiences have reported satisfactory and reproducible early and mid-term outcomes (2–5) in both standard and challenging clinical/anatomical scenarios, including emergency settings, cases involving previous aortic surgery and postdissection TAAAs, and those with hostile aortic-iliac anatomy (6–12).

The durability of renal, mesenteric, and celiac arteries [target arteries (TAs)] or stent-graft patency is one of the key factors contributing to the technical and clinical success of FB-EVAR procedures since the loss of these arteries can be life-threatening (13). Suppose it should be considered true for renal and celiac arteries (14–18). In that case, it becomes particularly important for the superior mesenteric artery (SMA) because the acute loss of this vessel causes a direct fatal event.

Previous experiences reported outcomes and risk factors for technical/clinical failure in managing renal and celiac arteries (13–18) during F/B-EVAR. However, this aspect has been rarely analyzed in previous literature studies dedicated to SMA, and few data are currently available.

The present study aimed to report and analyze the SMA-related outcomes of FB-EVAR to treat CAAAs and TAAAs.

## Methods

### Study design and patient selection

This single-center observational study was performed without funding from companies or other organizations and approved by the local review board (T.Ev.AAA-155/2015/U/Oss). All patients undergoing FB-EVAR (Cook Zenith platform, Cook Medical LLC, Bloomington, IN, USA) for CAAAs and TAAAs (degenerative or postaortic dissection) between 2016 and 2021 were prospectively grouped and retrospectively analyzed. FB-EVAR repair was proposed for patients with CAAAs or TAAAs, where standard endovascular endografting was not possible, at high risk for open repair if anatomically suitable (1). An infrarenal neck length <10 mm was usually adopted to indicate F/B-EVAR repair. Each patient signed dedicated informed consent for endovascular aortic repair and anonymous data analysis for retrospective clinical studies. According to the European General Data Protection Regulation (GDPR), all cases were deidentified with a coding number and clustered in an electronic database. Anatomical, procedural, and postoperative data were analyzed and reported.

### Endograft sizing and planning

Custom-made and off-the-shelf devices were used according to clinical and anatomical patient's characteristics. Patient-specific endografts were planned for elective cases by the same surgical team performing procedures and confirmed by the Cook Zenith Planning Center for fenestrated and branched endografts. Since 2012, the Cook Zenith off-the-shelf multibranched thoracoabdominal device (T-Branch) has been used for patients under emergencies (symptomatic, rupture, diameter >80 mm) or elective cases with anatomical feasibility and without adjunctive healthy aortic coverage other than a custom-made implant (7).

The proximal sealing zone was evaluated, measuring at least 2 cm the length of the healthy aortic wall (regular cylindrical shape—with no posterior bulging) in the multiplanar reconstructions. In this segment, a circumferential apposition between the endograft and aortic wall was expected (no scallop design in these 2 cm), and the main-body oversize was usually about 20%. TAs were analyzed (diameter and main trunk length) during preoperative computed tomography angiography (CTA) to select the most appropriate bridging stent-graft. The patency of the hypogastric artery was consistently preserved through endovascular (considered the primary choice) or surgical planned adjunctive maneuvers.

### Preoperative superior mesenteric artery evaluation

**Figure 1:**
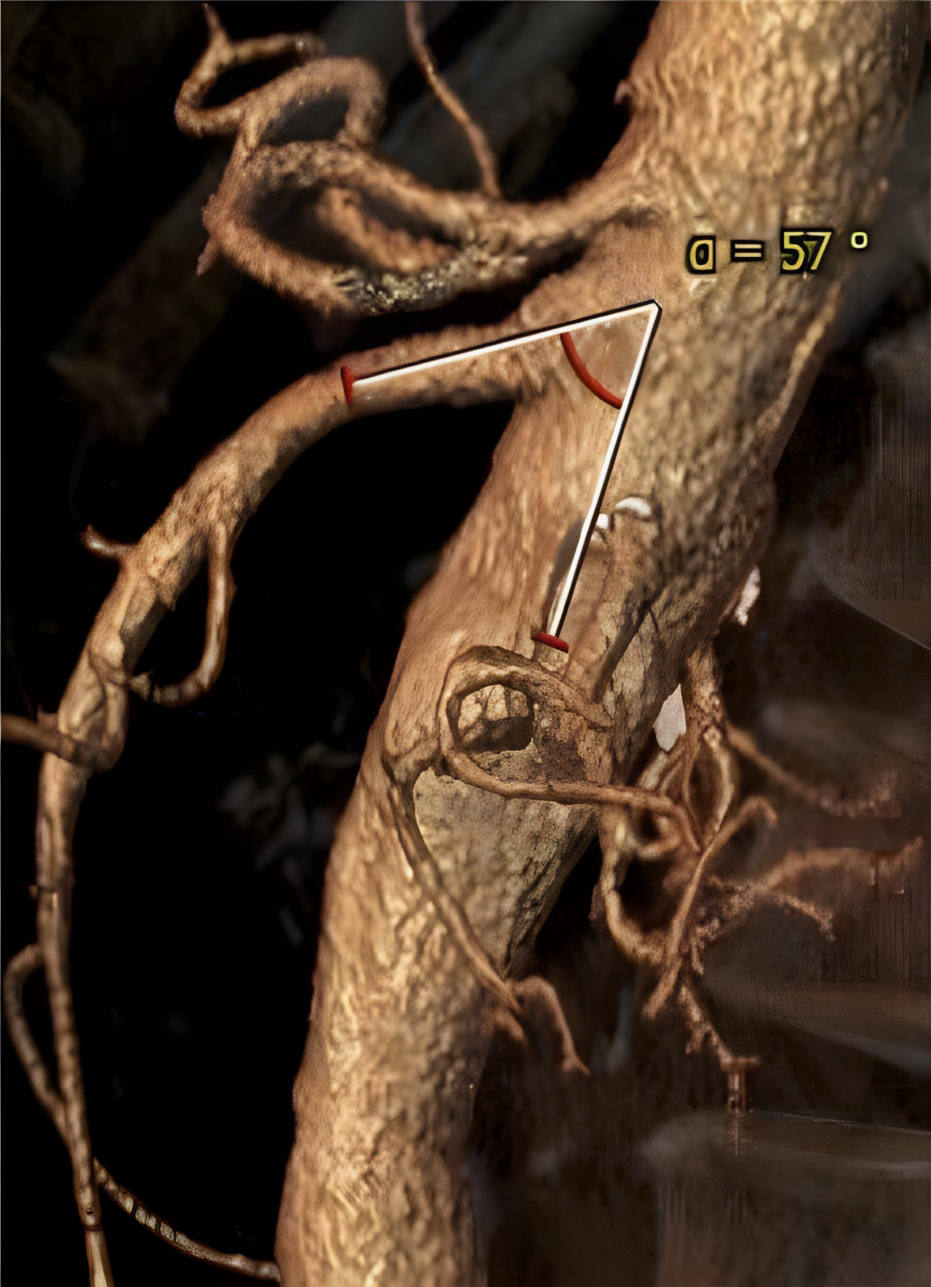
Volume rendering reconstruction of preoperative computed tomography angiography; angle between the longitudinal axis of the aorta and the main superior mesenteric artery trunk was evaluated by an electronic caliper provided by the 3-Mensio software.

**Figure 2:**
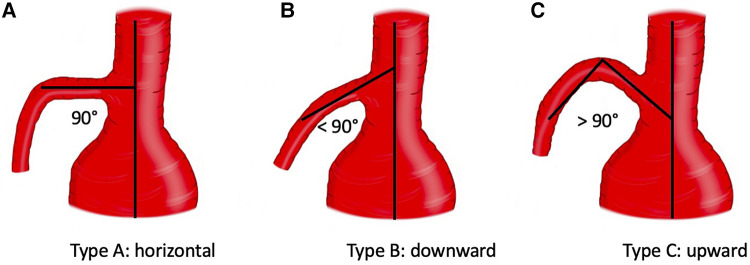
Configuration of superior mesenteric artery according to the orientation. Three types were identified: A (perpendicular), B (downward), C (upward).

Preoperative thoracoabdominal CTAs were retrospectively reviewed. Postprocessing evaluations were performed using dedicated software for advanced vessel analysis (3-Mensio, Vascular Imaging, Bilthoeven, The Netherlands). The main trunk length (linear distance between the SMA origin and the first branch) and diameter of the superior mesenteric artery were evaluated along with the presence of ostial stenosis, thrombosis, and calcification. The aneurysm diameter and aortic diameter at the SMA origin were also assessed. Using the electronic angular caliper provided by 3-Mensio software in the volume rendering reconstructions (Figure 1), the angle between the longitudinal axis of the aorta and the SMA main trunk was evaluated to define the SMA configuration, which was classified as perpendicular (A), downward (B), or upward (C) (Figure 2).

### Superior mesenteric artery incorporation

Superior mesenteric artery revascularization was performed by fenestration or external directional branch design according to the aortic diameter at the level of vessel's origin (1). Balloon-expandable stent-grafts were always used in fenestration design as bridging stents, while balloon- or self-expandable stent-grafts were used in branched design according to the TAs' anatomical characteristics and physicians’ preference. The length and diameter of the bridging stent-grafts were preoperatively evaluated according to the anatomical SMA features. Relining with bare metal stents (balloon- or self-expandable) was performed in case of residual stenosis/kinking of the bridging stent-graft, acute angle between the stent-graft and native vessels (not smooth/natural angle at the level of transition between the bridging stent-graft and the distal native vessel), or distal dissection of the arteries.

### Definitions and endpoints

Preoperative, intraoperative, and postoperative data, definitions, and outcomes were reported and classified according to the current Society of Vascular Surgery (SVS) reporting standard (1). Superior mesenteric artery-technical success (SMA-TS) and SMA-adverse events (SMA-AEs) were defined as primary outcomes of the study. Secondary outcomes were mortality and freedom from re-interventions (FFRs—overall and SMA-related) during the follow-up**.**

For the present study, SMA-TS was defined as successful SMA cannulation and stenting, SMA patency at completion angiography without SMA-related type I–III endoleaks, stenosis/kinking, dissection, rupture, and 24-h mortality. SMA-AE was defined as one among bowel ischemia (clinical or radiological manifestations), SMA-related stenosis, occlusion, endoleak, reintervention, and mortality.

### Follow-up

Laboratory evaluations of renal, hepatic, pancreatic function, and thoracoabdominal CTA were performed before discharge (19). The follow-up surveillance program consisted of Doppler ultrasound (DUS) or contrast-enhanced DUS (CEUS) at 6, 12 months, and yearly after that. In case of diagnostic doubts, a CTA was always performed. Patients received dual antiplatelet therapy from discharge to the first 6 postoperative months.

### Statistical analysis

Continuous variables were reported as means and standard deviations, while categorical ones were reported as numbers and percentages. Uni- and multivariate analyses were performed to evaluate potential risk factors for the study endpoints. Preoperative anatomical characteristics of the SMA and the aorta, endograft design (fenestration vs. external branch), setting of repairs (elective vs. emergency), and procedural data (fenestration/branch, number, and type of bridging stent-grafts, need of relining) were considered as risk factors for this analysis. Survival and FFR were evaluated using Kaplan–Meyer analysis. Statistical analysis was performed using SPSS 28.0 (SPSS statistical software, Chicago, IL, USA).

## Results

### Patient selection

In total, 228 consecutive patients underwent FB-EVAR for CAAAs and TAAAs. Among them, 14 (6%) patients were excluded for unavailability of preoperative CTA and 14 (6%) were excluded because the FB-EVAR implant did not require SMA incorporation. Finally, 200 (88%) cases met the study's inclusion criteria and were considered for the analysis. Of the 200, 17 (9%) patients were managed in an emergency clinical setting (rupture with stable hemodynamic parameters: 13; symptomatic: 4). The demographics, cardiovascular risk factors, and preoperative comorbidities of the 200 cases considered for the present study are summarized in Table 1.

**Table 1:** Demographics, cardiovascular risk factors, and preoperative comorbidities.

	*N*	%
Male	194	97
Hypertension	182	91
Smoke	154	77
Dyslipidemia	148	74
Diabetes	32	16
Chronic obstructive pulmonary disease	82	41
Coronary artery disease	77	39
Atrial fibrillation	23	12
Peripheral artery occlusive disease	24	12
Stroke	23	12
Body mass index >30	40	20
Chronic renal failure	85	43
Dialysis	3	2
Previous aortic surgery	64	32
American Society of Anesthesiologists score 3	72	36
American Society of Anesthesiologists score 4	128	64
	*N*	SD
Mean age (years)	73	5

### Preoperative aortic and SMA anatomical details

The indication for FB-EVAR repair was CAAAs, TAAAs, and a failed previous EVAR in 91/200 (45%), 101/200 (51%), and 8/200 (4%) cases, respectively. Sixty-four (32%) patients had a previous aortic surgery (open: 38, endovascular: 17, both open and endovascular: 9), and 7 (4%) were chronic postdissection TAAAs. Five (3%) patients had a history of previous perivisceral aortic repair, and there was no case of previous SMA stenting. The mean aneurysm diameter and aortic diameter at the SMA origin were 64 ± 13 mm and 33 ± 12 mm, respectively. The mean SMA diameter and main trunk length were 8 ± 1.5 mm and 43 ± 16 mm, respectively. There were 3/200 (1%) cases of severe (>50%) SMA ostial stenosis, and the aorta had thrombotic (>50% of circumference) apposition at the level of the SMA origin in 23/200 (12%) patients. The SMA configuration was A, B, and C in 63/200 (31%), 132/200 (66%), and 5/200 (3%) cases, respectively. No SMA was involved in the dissection or originating from the false lumen in chronic postdissection TAAAs.

### Endograft configuration

Custom-made and off-the-shelf devices were used in 140 (70%) and 60 (30%) patients, respectively. Endograft design with a fenestration branch, a directional branch, or both fenestration and directional branches was planned in 128/200 (64%), 60/200 (30%), and 12/200 (6%) cases, respectively. A superior mesenteric artery was incorporated using a fenestration in 131/200 (66%) cases, and a directional branch was incorporated in 69/200 (34%) cases. Directional branches were used more commonly to incorporate the SMA in patients with TAAAs [OR 12 (95% CI: 3.9–34.8), *P* < .001] or aortic diameter >35 mm at the level of SMA [OR: 5 (95% CI: 2.0–19.9), *P* < .001] and in patients needing ≥2 stents for SMA incorporation [OR: 8 (95% CI: 2.3–24.2), *P* = .001].

### Procedure

**Figure 3:**
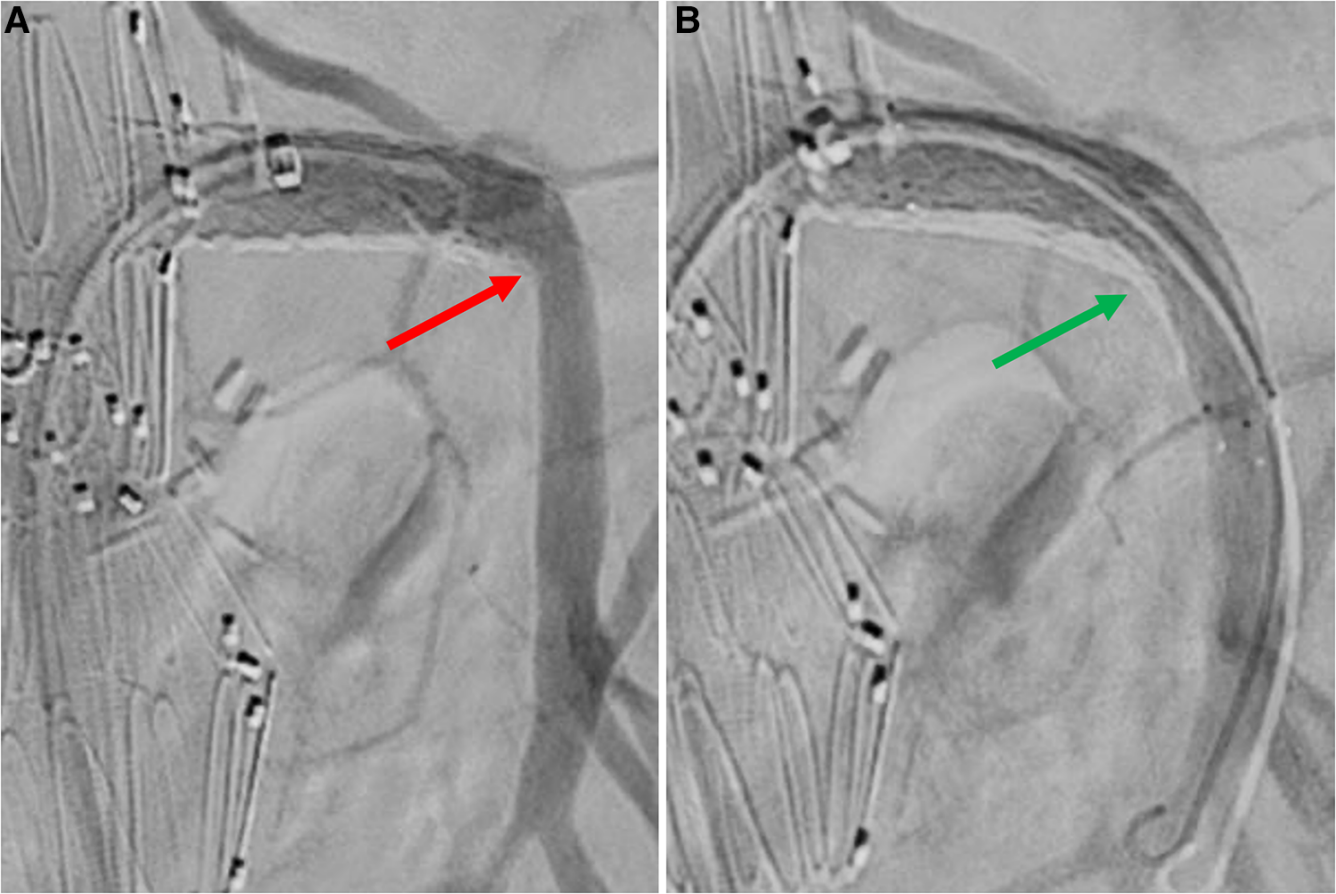
Selective angiography of superior mesenteric artery after bridging stenting. A: angiography without Rosen guidewire identifies an acute angle between SMA stent-graft and native vessel (red arrow). B: angiography without Rosen guidewire after relining by self-expandable bare metal stent does not identify any angle (green arrow).

**Figure 4:**
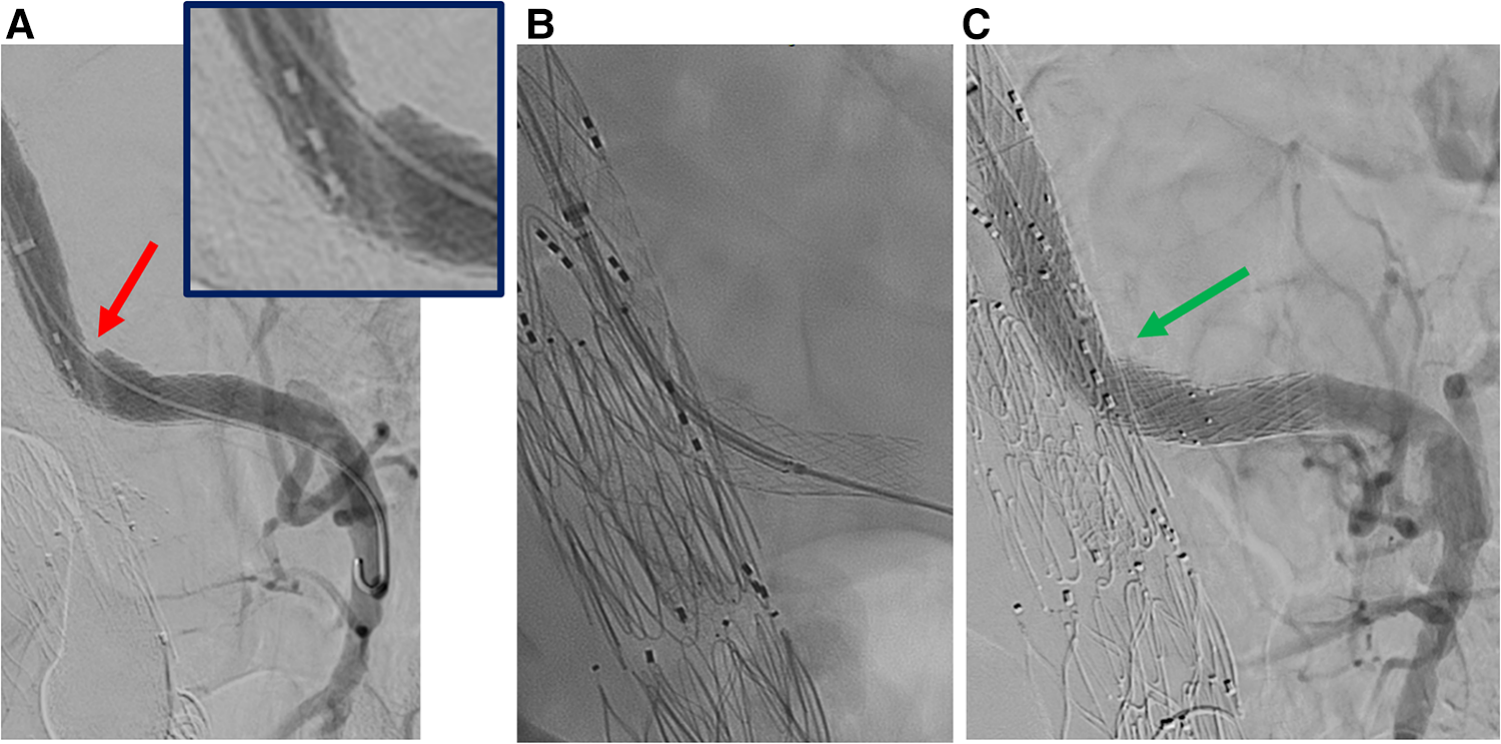
Selective angiography of superior mesenteric artery after bridging stenting. A: stenosis of the stentgraft (red arrow). B: relining by balloon expandable stent. C: angiography without Rosen guidewire after relining does not identify any stenosis (green arrow).

**Figure 5:**
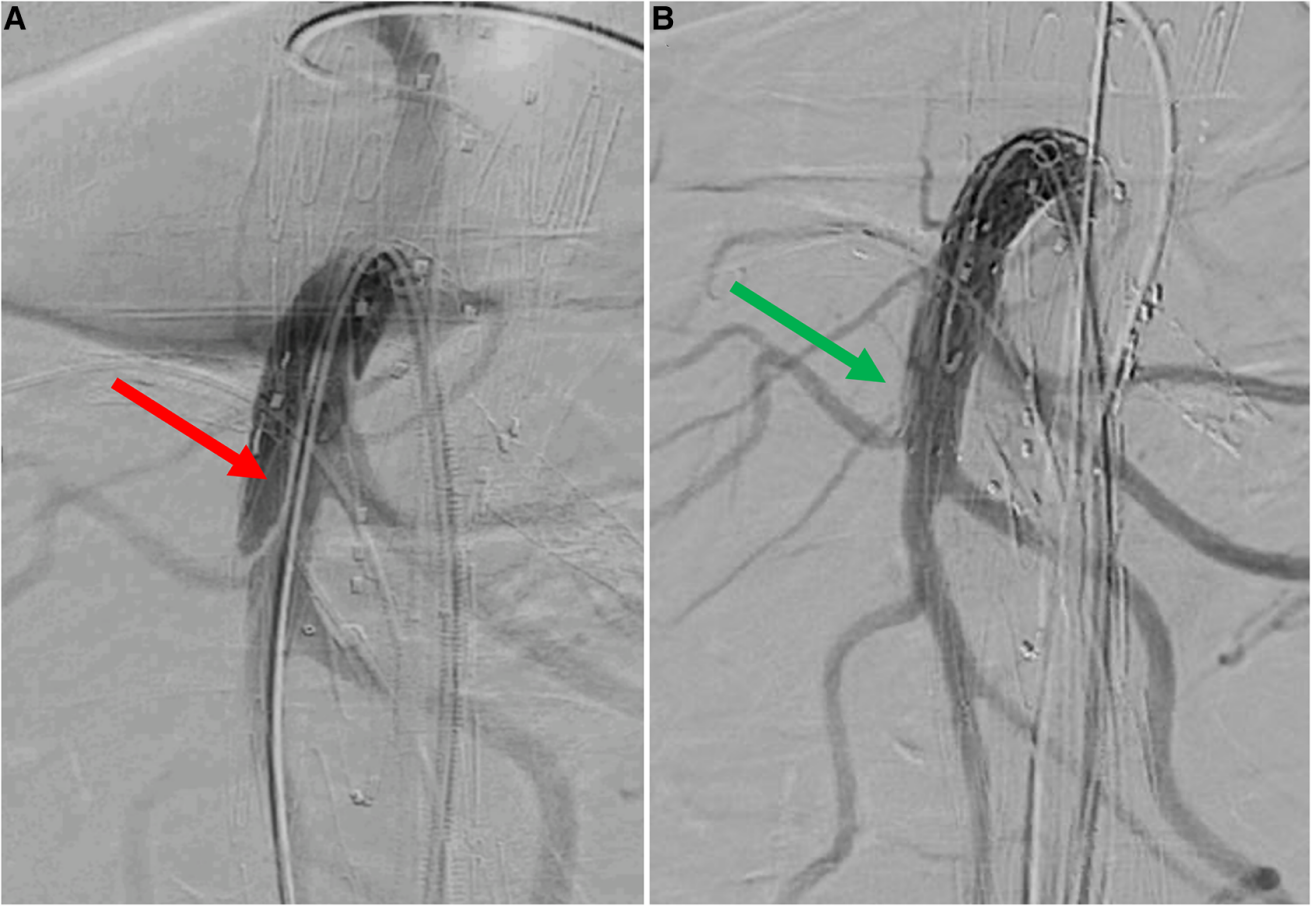
Selective angiography of superior mesenteric artery after bridging stenting. A: dissection of native superior mesenteric artery distally to the stentgraft (red arrow). B: angiography without Rosen guidewire after relining by self-expandable bare metal stent does not identify any defect (green arrow).

Balloon-expandable or a combination of balloon- and self-expandable stent-grafts were used as SMA bridging stent-grafts in 194/200 (97%) and 6/200 (3%) cases, respectively (Table 2). Two stent-grafts were necessary as bridging devices for incorporating the SMA in 44/200 (22%) patients (excluding relining by bare metal stents). Relining of the SMA stent-graft using bare metal stents was performed in 41/200 (21%) cases due to an acute angle between the stent-graft and native vessel (39 cases), stent-graft stenosis (1 case), or SMA dissection (1 case), as shown in Figures 3–5. In these cases, SMA relining was performed with a self-expandable bare metal stent in 40 cases and a balloon-expandable bare metal stent in 1 case. Type A or C SMA configuration was an independent risk factor for SMA stent-graft relining with bare metal stents [OR: 17 (95% CI: 1.8–157.3), *P* = .01]. For the superior mesenteric artery, technical success was achieved in all cases.

**Table 2:** Types of stent-grafts implanted as bridging stents in the superior mesenteric artery for fenestrated and branched endografts.

	*N*	%
Overall cases	200	100
Atrium Advanta	119	59
Bentley Begraft plus	3	2
Gore VBX	72	35
Atrium Advanta + Gore Viabahn	3	2
Gore VBX + Gore Viabahn	3	2

The mean procedural and fluoroscopy times were 325 ± 120 and 93 ± 20 min, respectively. The mean amount of iodinated contrast agent used was 185 ± 40 ml. At the end of the procedure, all patients were admitted to the intensive care unit (ICU) with a subsequent mean hospitalization in ICU of 24 ± 18 h.

### Early results

Five (2.5%) patients had postoperative clinical and radiological signs of bowel ischemia. In all cases, preoperative anatomical challenging characteristics (stenosis, calcification, thrombus) were noted, and no defect in the native SMA and SMA stent-graft patency (stenosis, occlusion, dissection) was detected at the postoperative CTA. Moreover, they had no defects in celiac trunk patency. Three of them required a bowel resection. In total, 32/200 (16%) patients required reinterventions within 30 days, which were mostly surgical access-related in 17/32 (53%) cases. Fourteen (7%) patients died during the hospitalization, with 10 (5%) events within 30 postoperative days. The causes of mortality are summarized in Table 3. Emergency cases [OR: 33 (95% CI: 5.7–191.3), *P* = .001], peripheral arterial occlusive disease [OR: 14 (95% CI: 2.3–88.8), *P* = .004), and bowel ischemia [OR: 41 (95% CI: 1.9–87.9), *P* = .01] were independent risk factors for 30-day/in-hospital mortality.

**Table 3:** Final causes of 30-day/in-hospital mortality.

	*N*	%
Cardiac morbidity	5	36
Cerebral hemorrhage	1	7
Hemorrhagic shock	1	7
Multiorgan failure/bowel ischemia	3	21
Pulmonary morbidity	4	29
Overall	14	100

### Midterm results

**Figure 6:**
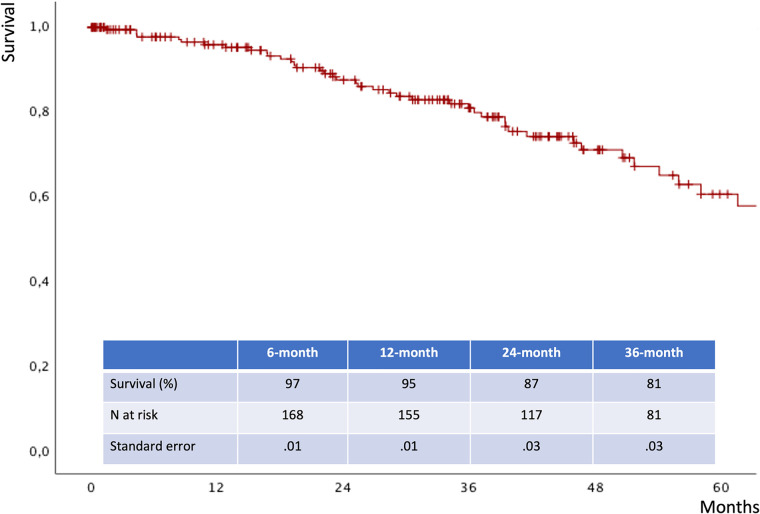
Follow-up survival estimated by Kaplan Meier analysis.

**Figure 7:**
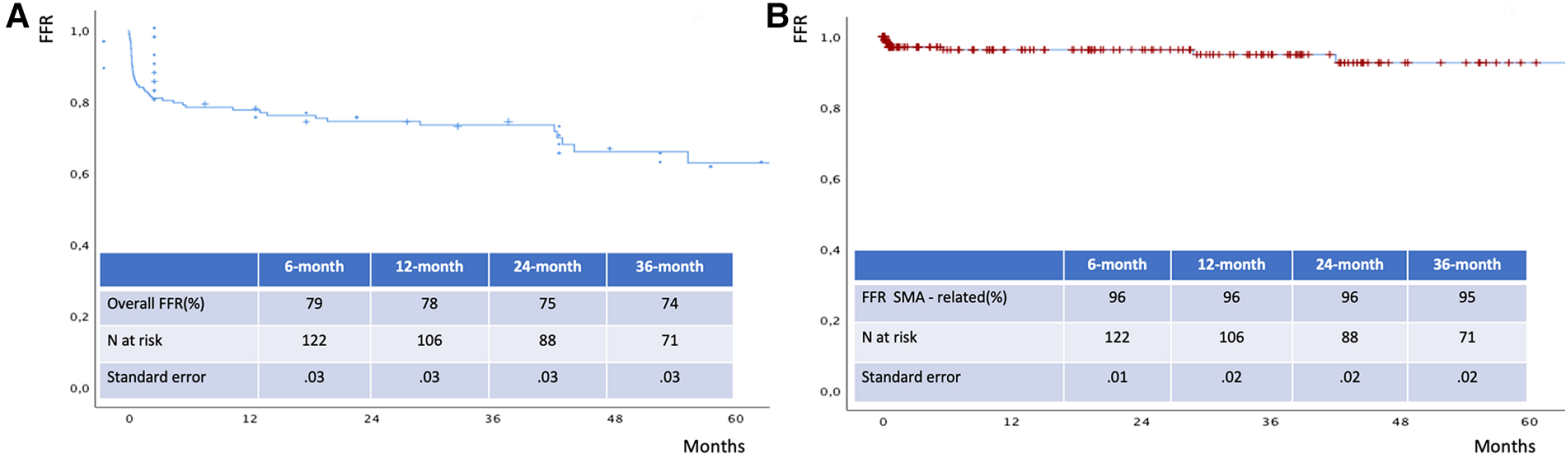
Freedom from overall (A) and SMA - related (B) reinterventions estimated by Kaplan Meier analysis. FFR: freedom from reintervention.

The mean follow-up time was 32 ± 24 months. The estimated 3-year survival was 81% (Figure 6), with no case of SMA-related mortality or occlusion at follow-up. The values for estimated 3-year freedom from overall and SMA-related reinterventions were 74% and 95% (Figures 7A,B), respectively. In total, 15 (7.5%) patients had SMA-AEs; 9 (60%) and 6 (40%) events occurred within 30 postoperative days and during the follow-up, respectively. They were classified into bowel ischemia (five cases), endoleaks (eight cases), and stent-graft stenosis/compression (two cases). Table 4 summarizes each of these patients, the timing of event occurrence, management, and the result. Aortic diameter ≥35 mm at the SMA origin was an independent risk factor for SMA-AEs [OR: 4 (95% CI: 1.4–13.8), *P* = .01].

**Table 4:** Superior mesenteric artery-adverse events: timing, management, and clinical outcomes.

*N*	Timing	SMA-AE	Management	Outcome
1	Within 30-day	Bowel ischemia	Left hemicolectomy	In-hospital mortality
2	Within 30-day	Endoleak Ic	Relining	Sealed
3	Within 30-day	Endoleak Ic	Relining	Sealed
4	Within 30-day	Stenosis	Relining	Solved
5	Within 30-day	Endoleak III	Relining	Sealed
6	Within 30-day	Bowel ischemia	Right hemicolectomy	In-hospital mortality
7	Within 30-day	Bowel ischemia	Left hemicolectomy	In-hospital mortality
8	Within 30-day	Bowel ischemia	Conservative	In-hospital mortality
9	Within 30-day	Bowel ischemia	Conservative	In-hospital mortality
10	After 30-day	Stenosis	Relining	Solved
11	After 30-day	Endoleak III	Relining	Sealed
12	After 30-day	Endoleak III	Relining	Sealed
13	After 30-day	Endoleak III	Relining	Sealed
14	After 30-day	Endoleak Ic	Relining	Sealed
15	After 30-day	Endoleak III	Relining	Sealed

SMA-AE, superior mesenteric artery-adverse event.

## Discussion

In this study, we have reported a single-center 6-year experience of 200 FB-EVAR procedures to manage CAAAs or TAAAs with a mean follow-up of 32 months. Results were satisfactory in terms of early postoperative morbidity, 30-day/in-hospital mortality, freedom from reinterventions, and survival during the follow-up. These outcomes are in line with the findings of previous single and multicenter studies conducted by European and US aortic centers over the last few decades (1–12). These results support the rationale behind the widespread use of FB-EVAR as the primary endovascular solution for CAAAs/TAAAs in high-risk patients with anatomical feasibility.

The technical and clinical success of FB-EVAR is strictly related to TAs' cannulation/stenting and to guarantee their patency during a life-long follow-up (13). Previous studies focused on intraoperative, early, or late occlusions of celiac and renal arteries, especially if the latter are incorporated using the directional branch design (14–18). Currently, comprehensive and dedicated data on SMA results are lacking, and this absence of data is relevant since most recent FB-EVAR experiences report a wide use of 3–4 fenestrated (or branched) endograft configurations (20). Even though these designs ensure a safe and reliable proximal sealing zone in CAAAs over long-term follow-up, they also may create potential and life-threatening complications in the case of serious SMA-related adverse events (20–22).

The present study aimed to report specific data about the SMA-related outcomes in FB-EVAR for CAAAs and TAAAs. Significant epidemiological and intraoperative information was discovered in the current analysis. The most frequent orientation of the SMA main trunk is perpendicular (type A—66%) or downward (type B—31%). In only 3% of cases, the orientation of the SMA was upward. It is an important detail to consider during endograft planning when choosing a fenestration or directional branch design for the SMA. In most cases, caudal direction branches can be safely utilized to allocate arteries oriented horizontally or in a downward direction. On the other hand, in the 3% of upward-oriented SMA cases, fenestrations or retrograde branches may be considered to facilitate SMA cannulation and stenting. However, fenestrations and branches were designed to accommodate 66% and 34% of SMA anatomies, respectively. Obviously, this result reflects our philosophy of endograft planning that prefers an external branch in the case of large aortic diameter at the level of the SMA to avoid a long gap distance between the hypothetical fenestration and the origin of vessels, reducing the possibility of TA instability during the follow-up. Once again, another finding from our statistical analysis confirms that a directional branch design for the SMA is more frequently adopted in patients with TAAAs (*P* < .001) or aortic diameter ≥35 mm at the SMA origin (*P* < .001) and those needing ≥2 stent-grafts (*P* = .001) as bridging stents for SMA.

As regards the choice of the bridging stent-graft, the most frequent option was the balloon-expandable type (97%), with only a few cases (3%) managed using a combination of balloon- and self-expandable stent-grafts and no cases managed with only a self-expandable stent-graft.

However, relining of the SMA stent-graft using a bare metal stent was reported in a not negligible rate of cases (21%). The main reason for relining was the correction of an acute angle between the stent-graft and the native SMA (95% of cases). Most of the relining was performed using self-expandable bare metal stents, which could be attributed to the high rate of balloon-expandable stent-grafts implanted. Moreover, SMA relining was associated with type A or C SMA configuration [OR: 17 (95% CI: 1.8–157.3), *P* = .01]. Unfortunately, the low rate of adverse events during the follow-up and the absence of a control group (acute angle without relining) did not allow for a subanalysis of the real efficacy of this adjunctive stenting. At the moment, it is an empiric adjunctive maneuver performed to correct a not ideal radiological image, and we have no data to confirm whether it is effective in the prevention of acute SMA stent-graft occlusions or stenosis during the follow-up. Nevertheless, no complications related to the relining stents were reported in either the procedural or follow-up results.

Overall, procedural results were excellent, with no case of intraoperative SMA-related technical failure. It seems obvious but is a crucial point in the FB-EVAR procedure because an acute intraoperative SMA loss is a lethal complication that should always be avoided. For this reason, it is mandatory to underline how all considerations about preoperative planning and sizing and procedural maneuvers (cannulation, manipulations, stent-grafting, and flaring) aim to achieve successful SMA management. The facilities of a modern hybrid room, such as intraoperative cone beam CT and intravascular ultrasound, are essential tools to be used in case of any diagnostic doubts to optimize the intraoperative control of quality (23, 24).

SMA patency is not the only aspect to evaluate for perioperative patient safety. We have reported five cases of bowel ischemia with SMA patency and the absence of any stent-graft defect at postoperative CTA. Three of these events were serious and required a bowel resection, while the remaining two cases were managed by conservative medical therapy. The origin of these events is probably multifactorial and can be explained by distal embolization during catheterization maneuvers, postoperative hypotensive status caused by other clinical postoperative complications, or multiorgan failure. It was an independent risk factor (OR: 41) for 30-day/in-hospital mortality as well as emergency TAAA repair (OR: 33) and preoperative PAOD (OR: 14). Overall, 30-day/in-hospital mortality was 7%; these data are in line with the most recently published FB-EVAR European and US experiences, and they can be considered satisfactory due to the presence of both emergency and elective repairs (1–12).

The estimated 3-year survival was 81%, which is comparable with the previous data available in the literature (1–12). There were no cases of aortic- or SMA-related deaths at the follow-up. Satisfactory results were also reported in terms of freedom from overall and SMA-related reinterventions, which were 74% and 95%, respectively.

In total, 15 (7.5%) patients had SMA-AEs: 60% in the postoperative period and 40% during the follow-up. They were caused by bowel ischemia (five cases), endoleaks (eight cases), and stenosis (two cases). As reported above, all cases of bowel ischemia occurred during the perioperative period and were not associated with defects in SMA patency. A surgical repair was required in three of five cases, and it had a negative impact on patient survival. Among the endoleak cases, three were detected at postoperative CTA and were successfully treated before discharge. They were not detected at the completion of angiography, but they should probably be considered a suboptimal technical result. The other five endoleak cases were detected during the follow-up (four in routine tests and one in a symptomatic patient) and were successfully managed by stent-graft relining. Both SMA stent-graft compressions were detected (one early and one at follow-up) at CTA and managed by an adjunctive balloon-expandable stent-graft. It is important to underline that biplanar intraoperative angiography may underestimate these findings. Therefore, it is crucial to emphasize the importance of dedicated intraoperative imaging tools for high-quality control. Interestingly, there was no case of native SMA stenosis distally to the bridging stent-graft or stent-graft fracture. An important finding in our analysis was that aortic diameter ≥35 mm at the level of the SMA was an independent risk factor for SMA-AEs.

Several limitations must be considered in the present study. First, it is a single-center, retrospective study with a relatively small cohort of patients and mid-term follow-up. However, it should be considered that it is one of the largest single-center series reported in the last years and FB-EVAR is a relatively new technology with no big data about long-term follow-up. The small sample size may be associated with a theoretical statistical type B error, reducing the strength of study's conclusions. Second, the operator's learning curve was not considered, and it is crucial to optimize technical and clinical results in these challenging cases. In our department, the FB-EVAR program started in 2010, and the patients included were treated after 5 years of experience by using well-standardized pre-, intra-, and postoperative protocols in a hybrid room with all the available facilities (vessel navigator, CO_2_ angiography, cone beam CT, and IVUS). Third, the protocol of home surveillance consists of different imaging modalities (DUS, CEUS, and CTA) with different sensitivity and specificity levels to detect TVV-related endoleaks, stent-graft stenosis/kinking, or other complications. This may be reason for a part of undetected or underestimated adverse events during the follow-up and the subsequent underestimate of the rate of SMA-related reinterventions. Fourth, a dedicated analysis of stent-grafts of different brands used to incorporate the SMA was not performed because the number of cases was too small to guarantee a significant subgroup analysis. We report data dividing SMA managed by SE or a combination of SE  and BE stent-grafts with similar results in terms of SMA-AEs and follow-up patency. Finally, it is impossible to exclude that acute SMA thrombosis was the real cause of death during the follow-up in cases of unknown cause of mortality.

## Conclusion

The orientation of the superior mesenteric artery determines the necessity of stent-graft relining. Aortic diameter >35 mm at the level of the SMA is a predictor of SMA-AE. However, SMA-related outcomes of FB-EVAR are satisfactory, with excellent technical success and encouraging clinical outcomes during the follow-up.

## Data Availability

The original contributions presented in the study are included in the article/Supplementary Materials, further inquiries can be directed to the corresponding author.
